# Comprehensive Behavioral Therapy of Trichotillomania: A Multiple-Baseline Single-Case Experimental Design

**DOI:** 10.3389/fpsyg.2020.01210

**Published:** 2020-06-10

**Authors:** Gioia Bottesi, Allison Jane Ouimet, Silvia Cerea, Umberto Granziol, Eleonora Carraro, Claudio Sica, Marta Ghisi

**Affiliations:** ^1^Department of General Psychology, University of Padova, Padova, Italy; ^2^Centro di Ateneodei Servizi Clinici Universitari Psicologici (SCUP), University of Padova, Padova, Italy; ^3^School of Psychology, University of Ottawa, Ottawa, ON, Canada; ^4^Department of Health Sciences, University of Firenze, Firenze, Italy

**Keywords:** Trichotillomania, Comprehensive Behavioral Model, single-case experimental design, self-monitoring, psychological treatment

## Abstract

Despite rapidly increasing knowledge about Trichotillomania (TTM), no gold-standard evidence-based psychological intervention has been identified. In the current study, we evaluated the potential efficacy of an eight-session psychological intervention for TTM, namely the Comprehensive Behavioral Model (ComB) treatment, using a multiple-baseline single-case experimental design with three Italian women with TTM. The study included three phases: baseline, intervention, and 3-month follow-up. We assessed the intervention using daily self-monitoring of number of hair-pulling episodes, number of pulled hairs per episode, degree of resistance to pulling urges, and degree of associated distress. We also assessed for reliable improvement in general distress from baseline to post-intervention. All participants completed treatment and showed improvements on those symptom measures that were most relevant to their individualized case conceptualization. However, no participants recovered completely or demonstrated reliable improvement in general distress. Our results provide initial evidence for the utility of the ComB treatment for TTM in an Italian clinical setting. Furthermore, they support the delivery of individualized and flexible psychological treatments when treating TTM.

## Introduction

Trichotillomania (TTM) is currently classified within the Obsessive-Compulsive and Related Disorders in the Diagnostic and Statistical Manual of Mental Disorders-Fifth Edition [DSM-5; American Psychiatric Association (APA), 2013]. People with TTM demonstrate recurrent hair-pulling resulting in hair loss; repeated attempts to decrease or stop the behavior; and clinically significant distress or impairment in social, occupational, or other important areas of functioning due to the behavior [American Psychiatric Association (APA), 2013]. Although much has been learned about TTM over the last decade, a gold-standard evidence-based psychological intervention for this disorder has not yet been identified. To date, treatments based on cognitive behavior therapy (CBT) have been the most tested and are thus the most empirically validated, with researchers reporting promising results about their effectiveness (e.g., Rehm et al., 2015a). Available psychological treatments for TTM include habit reversal therapy (HRT; Azrin and Nunn, 1973), acceptance and commitment therapy-enhanced HRT (Woods et al., 2006), dialectical behavior therapy-enhanced HRT (Keuthen et al., 2012), and interventions based on the Comprehensive Behavioral Model (ComB; Falkenstein et al., 2016).

The Comprehensive Behavioral Model (Mansueto et al., 1997) is a theoretical model that outlines the different factors (defined as “modalities”) maintaining individuals’ hair-pulling. These modalities include sensory (tactile, visual, or oral triggers), cognitive (thoughts that cause, facilitate, or maintain hair-pulling), affective (emotional states, such as anger, tension, or anxiety), motor (facilitative postures and motor habits), and place (external environmental cues, such as particular places, situations, or objects). Research findings suggest that psychological treatments for TTM may be more effective when they target the individual’s specific hair-pulling experience (e.g., O’Connor, 2002). Consequently, case conceptualizations consistent with the individual’s specific hair-pulling antecedents, consequences, and modalities may facilitate individualized psychological treatment for specific patients and hair-pulling episodes (Mansueto et al., 1999; Falkenstein et al., 2016). Furthermore, this case conceptualization could target both “automatic” (pulling without full behavioral awareness, typically during idle moments) and “focused” (intentional pulling to regulate intense emotions or unpleasant internal experiences) hair-pulling episodes (Mansueto et al., 1999). Given findings supporting hair-pulling as a dysfunctional emotion regulation strategy that people use to avoid, modulate, or alleviate negative emotions (e.g., Roberts et al., 2013), it is important to pay particular attention to the affective modality.

Falkenstein et al. (2016) provided promising preliminary data supporting the validity, feasibility, and acceptability of the ComB treatment. Specifically, they found that ComB led to reduced TTM symptom severity and impairment, and improved quality of life. Indeed, 38% of participants at post-treatment (after 12 weeks) and 11% of participants at 3-month follow-up met the criteria for clinically significant improvement. Despite its current use, the ComB approach and its efficacy still need systematic testing, with randomized controlled trials (RCTs) as paramount. Indeed, to date, a few RCTs have been conducted by different research groups in the United States (e.g., Woods et al., 2006; Franklin et al., 2011; Keuthen et al., 2012), Iran (Shareh, 2018), and the Netherlands (Keijsers et al., 2016), but none of them specifically tested the efficacy of the ComB treatment. To note, a research project entitled “Randomized Controlled Trial of the Comprehensive Behavioral Model for Trichotillomania” is currently underway in the United States^1^.

To date, no Italian treatment studies for TTM have been conducted. Although some RCTs have been carried out in other countries, testing the cross-cultural validity of empirically validated protocols is an important and clinically relevant endeavor. Indeed, cross-cultural differences may shape some TTM phenomenology, which may require that treatment protocols be adapted to meet culture-specific needs (Bottesi et al., 2016a). In this regard, previous research within Italian samples suggests that many phenomenological features of TTM among Italian individuals correspond to those of American individuals; however, there are also some notable differences (Ghisi et al., 2013; Bottesi et al., 2016a,b). For example, Italian individuals with TTM report pulling hair occurs both when alone and in the presence of others, whereas some findings within American samples suggest that the majority of pulling occurs when alone. Moreover, higher rates of trichophagia were observed in Italian individuals with TTM compared with rates reported in clinical samples from the United States (Bottesi et al., 2016b). Moreover, Italian and American clinical samples differ with respect to some affective correlates of TTM across the pulling cycle, for example, Italian individuals with TTM reported no significant variations in guilt, a result inconsistent with literature from American samples (Bottesi et al., 2016b).

Unfortunately, there are still some important barriers to recruiting large, homogenous samples necessary for RCTs in Italy. TTM remains poorly recognized and often inadequately treated in Italian clinical settings (Bottesi et al., 2016b, 2019). Specifically, the secretiveness and shame still associated with hair-pulling often leads to underreporting (e.g., Duke et al., 2010; Ghisi et al., 2013). Moreover, past negative treatment experiences erode people’s willingness to seek help (Stolzenburg et al., 2019) and appear to foster both their belief that TTM is incurable (Bottesi et al., 2019) and a current distrustful attitude toward mental health professionals (Franklin et al., 2008; Bottesi et al., 2016a,b). Taken together, the current state of research and clinical practice relevant to TTM in Italy suggests that the most viable stage of intervention implementation research to test the efficacy of psychological treatments targeting TTM in Italy is currently single-case experimental designs (SCEDs).

Single-case experimental designs are a valuable approach during the early stages of testing a psychological treatment’s efficacy, especially when (a) the clinical features of a condition reduce the feasibility of performing RCTs and (b) the theoretical framework underlying a treatment model aims to maximize the individualization of the intervention to each patient’s specific psychological functioning (Blampied, 1999; Kazdin, 2011). Since TTM is a heterogeneous disorder (e.g., Grant, 2015), sufferers vary in pulling sites, pulling styles, motivational factors underlying the behavior, and modalities, limiting the degree to which large treatment samples could be considered homogeneous. SCEDs focus on intraindividual change rather than change in the group aggregate. This methodology is highly compatible with clinical practice, and the frequent measurement inherent in the design facilitates assessing systematically both the degree and the pattern of change (Borckardt et al., 2008). In the current pilot feasibility study, we sought to evaluate the potential efficacy of an eight-session ComB treatment for TTM (Mansueto et al., 1999; Falkenstein et al., 2016; Bottesi et al., 2019) using a multiple-baseline SCED with three Italian women with TTM.

## Materials and Methods

### Design

We used a multiple-baseline SCED over three phases: baseline, intervention, and follow-up. Participants were randomly assigned to different baseline durations: 7, 14, and 21 days. Follow-up was set at 3 months following the intervention.

### Participants

Three women reporting clinically significant hair-pulling entered the study. Inclusion criteria were a diagnosis of TTM and minimum age of 18 years; exclusion criteria were current or past schizophrenia spectrum or other psychotic disorder, severe personality disorder, major neurocognitive disorder, and intellectual disability. All clinical diagnoses were established by a PhD-level clinical psychologist (Bottesi; see Intervention section below) using the Structured Clinical Interview for DSM-5 (SCID-5; First et al., 2015) and the Structured Clinical Interview for DSM-5 Personality Disorders (SCID-5-PD; First et al., 2016). All participants completed the study, and none were taking medication at the time of the intervention.

### Clinical Details

Participant 1 was a 23-year-old, unemployed woman whose TTM had been present for 13 years. She had a comorbid DSM-5 diagnosis of obsessive-compulsive personality disorder. She started pulling her eyebrows and eyelashes in response to sensations of itch and sticky lashes caused by conjunctivitis. She reported that, over time, she began pulling all the hairs she perceived as “not just right” and performing trichophagia (i.e., eating her hair) as an oral ritualistic behavior. Hair-pulling occurred primarily when she was speaking on the phone or watching TV. She reported that episodes occurred more frequently during stressful periods and when she felt angry with significant others. Hair loss was not visible due to make up and false eyelashes. She reported that she learned, over time, to accept the idea of living with TTM. She reported that, at age of 12 years, she attended three sessions with a counselor to treat her TTM; in her opinion, the professional was not willing to directly target her primary problem, therefore she dropped out.

Participant 2 was a 33-year-old woman whose TTM had been present for 21 years. She had a current comorbid DSM-5 diagnosis of major depressive disorder (mild severity). She was married and had three children. She was a university student and, due to financial problems, also had several temporary jobs. She reported that she started pulling her hair after experiencing sexual harassment at age of 12. She pulled hair primarily from her scalp and occasionally from her pubic area. She performed the behavior intentionally when she felt particularly anxious or sad. Her scalp was almost completely bald, which she attempted to disguise by wearing a bandana, which contributed to feelings of extreme shame and avoidance of social situations. She believed she would never feel better and that TTM was the worst thing that had happened in her life. She reported that she had accessed four psychologists and a psychiatrist, but no one provided a diagnosis or an effective treatment.

Participant 3 was a 22-year-old female university student whose TTM had been present for 9 years; she had no DSM-5 comorbid diagnosis. She pulled exclusively from her scalp any hair that she perceived as thick and coarse. She reported that pulling episodes occurred while watching TV and during study sessions; she pulled more frequently and a higher number of hairs when she was studying for exams. Hair loss was not visible, but she was worried that she might have lost control over the behavior, thus further increasing both the frequency of episodes and the amount of pulled hair. She reported no previous treatment experiences.

### Measures

The primary outcome measure was assessed *via* daily self-monitoring of pulling episodes from baseline until the end of the 3-month follow-up period. Daily measures were number of hair-pulling episodes; number of hairs pulled during each episode; the degree of resistance to pulling urges, from 0 (“no attempt to inhibit/interrupt the behavior”) to 10 (“successful attempt to inhibit/interrupt the behavior”); and the degree of associated distress, from 0 (“no distress at all”) to 10 (“extreme distress”). These measures mirror the DSM-5 diagnostic criteria for TTM [American Psychiatric Association (APA), 2013].

As a secondary outcome measure, participants completed the Italian version of the Depression Anxiety Stress Scales-21 (DASS-21; Bottesi et al., 2015) as an established measure of general distress (see, for example, Osman et al., 2012; Bottesi et al., 2015). The DASS-21 is a 21-item self-report questionnaire assessing symptoms of depression, anxiety, and stress over the previous week on a 4-point Likert-type scale. In the Italian validation, the factor structure provided evidence that the total score is a reliable measure of general distress. The total score demonstrated excellent internal consistency (non-clinical sample: *α* = 0.90 and clinical sample: *α* = 0.92), good test-retest reliability (*r* = 0.74), and adequate convergent and divergent validity (Bottesi et al., 2015).

### Procedure

Participants responded to announcements in local newspapers and in university buildings. A PhD-level clinical psychologist (Bottesi) trained in CBT conducted a preliminary interview with interested participants to assess their eligibility. Subsequently, they were provided with information about the program and the psychological intervention. Participants completed the DASS-21, and then received a record sheet and detailed written instructions to monitor daily their hair pulling episodes. The intervention was then scheduled according to the established individualized baseline durations. Treatment was administered by the same clinical psychologist and it was delivered at the Cognitive and Behavioral Therapy Service, a specialized university center. The study was conducted in accordance with the Declaration of Helsinki and it was approved by the Ethics Committee of Psychological Sciences of the local university. All participants provided written informed consent and free therapy was the only compensation.

### Intervention

The psychological intervention consisted of eight weekly sessions. Each session lasted 1.5 h. All three participants received the same treatment protocol; however, the specific content of each session was unique and based on the individualized case conceptualization. The content of these sessions was drawn directly from the 12-session treatment provided by Falkenstein et al. (2016); however, because we were required to follow the policies of the Cognitive and Behavioral Therapy Service (e.g., eight-session maximum), Sessions 1–5 of the original treatment were compressed and presented in a single session (Session 1). Session 1 included psychoeducation about TTM and the collaborative identification of the main modalities maintaining each patient’s (i.e., individualized) hair-pulling episodes. The contents of this session mirrored those of the original Section 1 (“Assessment and Functional Analysis”; four sessions) and Section 2 (“Identification and Targeting of Modalities”; one session). However, it is important to note that that some topics included in Section 1 (i.e., discussing motivation, functional analysis, and self-monitoring of pulling episodes) of Falkenstein et al. (2016) had already been introduced to each participant during the preliminary interview. Moreover, the study design we adopted enabled participants to become familiar with self-monitoring and to increase their awareness of their pulling modalities prior to Session 1. Because all participants found the self-monitoring feasible, acceptable, and useful, it was possible to effectively identify and target modalities during a single session (Session 1). The contents of Sessions 2–8 overlapped directly with those of the original Section 3 (“Identification and Implementation of Specific Interventions”; four sessions) and Section 4 (“Evaluation, Modification, and Relapse Prevention”; two sessions). Session 2 focused on stimulus control strategies and habit reversal training to target sensory, motor, and place modalities (Azrin and Nunn, 1973). Sessions 3–5 focused on cognitive restructuring and behavioral experiments to target: (1) dysfunctional beliefs about TTM and (2) general dysfunctional beliefs that caused significant distress/negative affective states, to help generalize the learning to everyday situations and reduce the use of hair-pulling to regulate emotions. Session 6 focused on teaching common stress-management techniques, such as progressive muscle relaxation (Jacobson, 1977) and breathing exercises (Andrews et al., 2003) to target the affective modality. Session 7 focused on relapse prevention, and Session 8 focused on review and consolidation. Specific between-session exercises were assigned at the end of each session. The content of any new between-session exercises was related to the main topics addressed during that session; participants received a handout summarizing the session contents at the end of each session. At the beginning of each session, the psychologist reviewed the between-session exercises with the participant. Participants completed the DASS-21 a second time at Session 8 (i.e., post-treatment).

### Statistical Analyses

**Figure 1:**
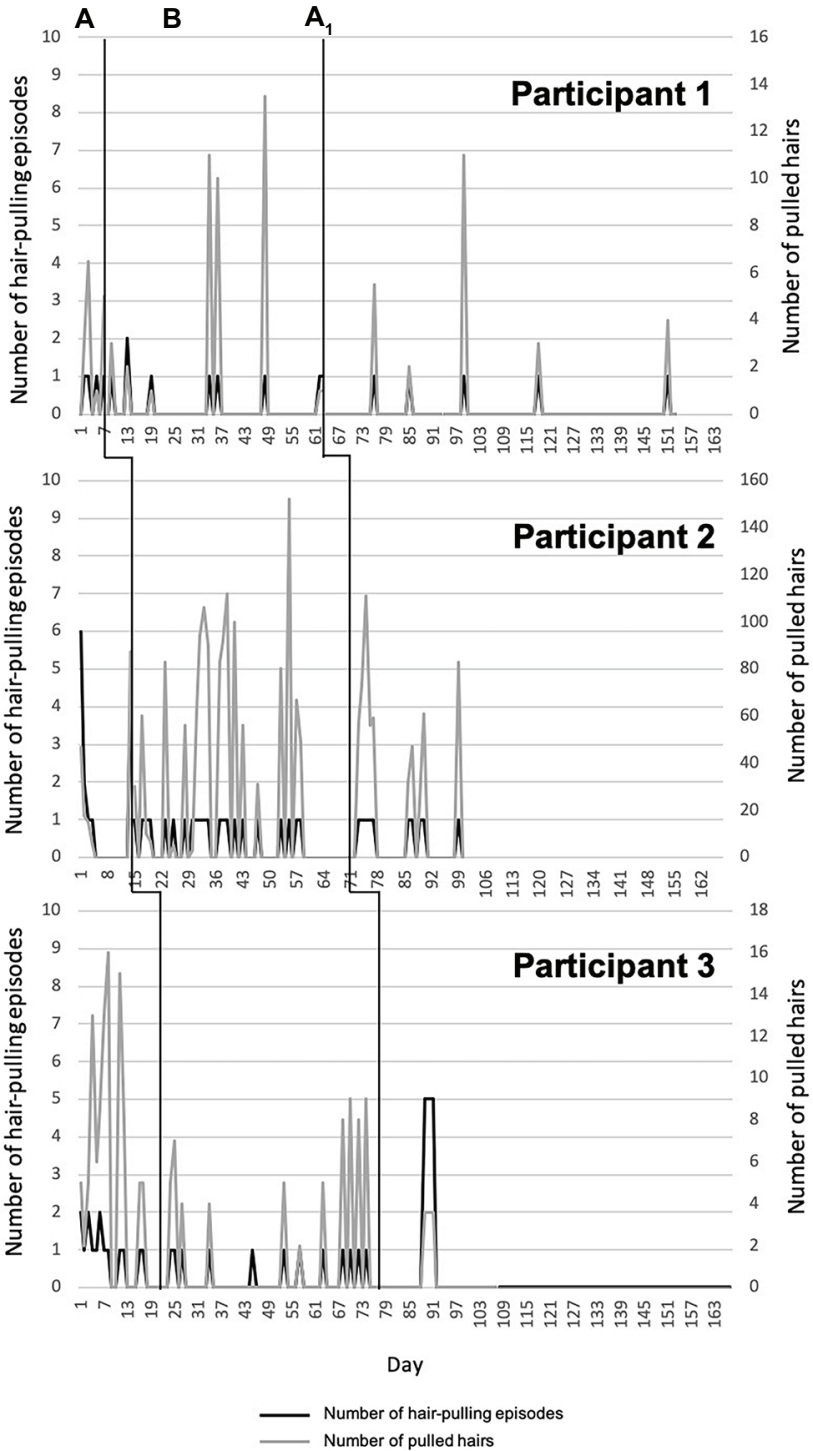
Number of hair-pulling episodes and number of pulled hairs over time (days).

**Figure 2:**
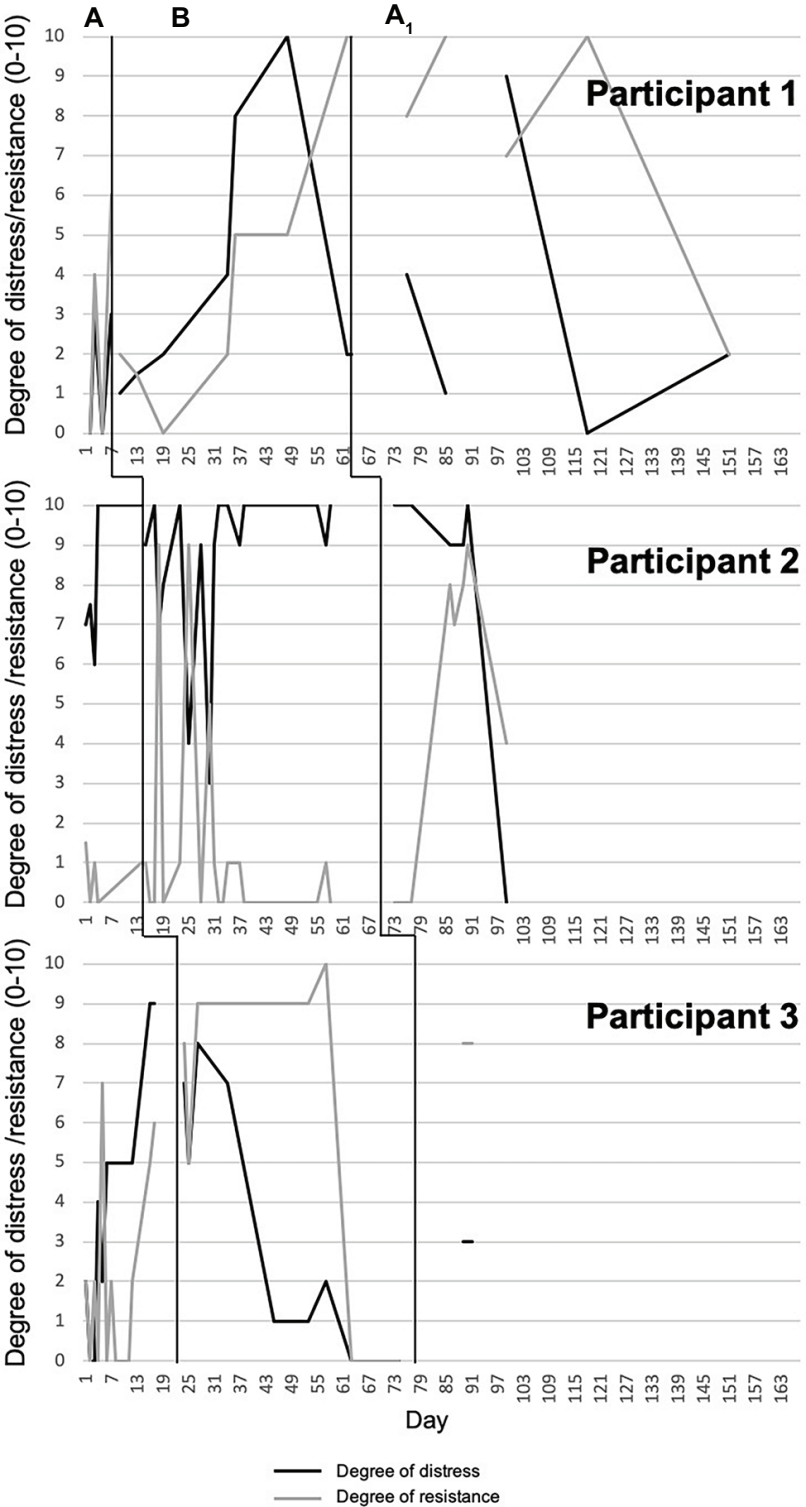
Degree of resistance to pulling urges and degree of associated distress over time (days).

Data from daily monitoring report sheets were graphed using Excel and interpreted by visual analysis of the graphs (Kazdin, 2011). We created one graph per participant for each symptom measure across all phases, based on each participant’s responses on the daily report sheets, ordered from shortest to longest baseline duration (see Figures 1, 2). We computed the Tau-U statistic (Parker et al., 2011) to test for data non-overlap between phases. Non-overlap is defined as “the separation of two ‘data clouds,’ giving equal attention to all data points” (Parker et al., 2011, p. 285). Greater difference scores between phases reflect greater non-overlap. In other words, if there is non-overlap, any changes observed in the target behavior in a given phase are not accounted for by the pattern of the same target behavior in the previous phase. Tau-U also corrects for any baseline trends. As such, it controls for any increase or decrease in the target behavior during the baseline phase that may continue into the treatment phase and thus be responsible for any change that one can attribute to the intervention. Taken together, by calculating the Tau-U statistic, we tested whether patterns of change in a given phase were likely attributable to the effects of that particular phase (i.e., attributable to the intervention). In the present study, we compared baseline vs. treatment and treatment vs. follow-up within each participant for each of the daily monitored symptom measures, using the Tau-U statistic. Furthermore, we calculated an aggregate value that reflects non-overlapping between phases across all cases (Parker et al., 2011).

To assess whether DASS-21 scores changed over time, we used an Excel spread sheet (i.e., the Leeds Reliable Change Indicator; Morely and Dowzer, 2014). We used the mean (clinical group: *M* = 21.1, *SD* = 12.1; non-clinical group: *M* = 12.3, *SD* = 8.3) and test-retest values reported in the Italian validation of the questionnaire (Bottesi et al., 2015) as criterion variables.

## Results

### Number of Hair-Pulling Episodes per Day

Participant 1 showed very little variability in number of hair-pulling episodes during baseline. In the initial weeks of treatment, she reported an initial increase followed immediately by a reduction, which was maintained during follow-up. Participant 2 showed high variability in number of hair-pulling episodes during baseline, followed by a clear reduction with a pronounced change in slope during treatment. This reduction was maintained during follow-up. Participant 3 demonstrated a decrease in number of hair-pulling episodes during baseline; this reduction was maintained through treatment. A few weeks into follow-up, she reported an increase in hair-pulling episodes, which was immediately followed by a maintained reduction for the remainder of the phase. Only Participant 2 showed a statistically significant change in level of non-overlapping data between baseline and treatment. There was no significant difference between treatment and follow-up for all participants except Participant 3 (Figure 1, Table 1).

**Table 1:** Tau-U calculations across phases for each participant.

	A vs. B + trend B − trend A				B vs. A_1_ + trend A_1_ − trend B			
Participant	Tau-U	SD Tau	*Z*	*p*	Tau-U	SD Tau	*Z*	*p*
Participant 1	−0.02	6.90	−0.15	0.89	0.00	7.48	0.00	0.99
Participant 2	−0.14	29.45	−1.70	0.007	−0.06	25.69	−1.25	0.41
Participant 3	−0.06	22.91	−0.74	0.49	0.34	13.86	2.60	0.01
Weighted average	−0.09	0.10	−0.82	0.41	0.17	0.15	11.34	0.26

Daily measure of symptom severity: number of hair-pulling episodes.

A, baseline; B, treatment; A_1_, follow-up. The values in bold refer to significant changes in level of non-overlapping data between phases.

### Number of Pulled Hairs per Episode

Participants 1 and 2 showed high variability across all three phases for number of pulled hairs during each episode. For Participant 1, the number of pulled hairs (eyelashes) was generally low, and there were no significant differences between phases. Participant 2 pulled a very high number of hairs across phases and demonstrated a statistically significant increase in number of hairs pulled between baseline and treatment. There was a slight reduction during follow-up, but no change in non-overlapping data between treatment and follow-up. Participant 3 showed high variability in number of hairs pulled per episode during baseline and a clear reduction with a pronounced change in slope during treatment and follow-up. In both cases, there was a significant change in level of non-overlapping data between phases (Figure 1, Table 2).

**Table 2:** Tau-U calculations across phases for each participant.

	A vs. B + trend B − trend A				B vs. A_1_ + trend A_1_ − trend B			
Participant	Tau-U	SD Tau	*Z*	*p*	Tau-U	SD Tau	*Z*	*p*
Participant 1	−0.05	14.25	−0.21	0.83	0.15	16.19	0.74	0.46
Participant 2	0.32	47.94	2.34	0.02	−0.18	61.61	−1.46	0.14
Participant 3	−0.01	39.66	−0.91	0.02	−0.52	19.82	−2.77	0.01
Weighted average	0.10	0.11	0.90	0.37	−0.27	0.16	−16.79	0.09

Daily measure of symptom severity: number of pulled hairs during each episode.

A, baseline; B, treatment; A_1_, follow-up. The values in bold refer to significant changes in level of non-overlapping data between phases.

### Degree of Resistance to Pulling Urges

Participant 1 showed high variability in resistance to pulling urges during baseline. There was a notable increase during treatment, whereas follow-up was characterized by more variability, with strong resistance reported early on, and lower resistance reported near the end of the phase. A trend toward a significant change in level of non-overlapping data between baseline and treatment emerged; no other differences were evident. Participant 2 demonstrated small and relatively stable resistance to pulling urges during baseline. Variability was greater during treatment, with initial high resistance followed by a decrease to small and stable resistance. Reported urge resistance remained low during the first week of follow-up and then increased for a few weeks before decreasing again to a level of 4. A trend toward a significant change in level of non-overlapping data between treatment and follow-up phases emerged. Participant 3 exhibited high variability in resistance to pulling urges during baseline. During treatment, resistance was high and relatively stable for the first several weeks, followed by a sudden decline to zero for the final 12 days. In follow-up resistance was high and stable. No significant difference between phases for Participant 3 emerged (Figure 2, Table 3).

**Table 3:** Tau-U calculations across phases for each participant.

	A vs. B + trend B − trend A				B vs. A_1_ + trend A_1_ − trend B			
Participant	Tau-U	SD Tau	*Z*	*p*	Tau-U	SD Tau	*Z*	*p*
Participant 1	0.39	14.35	1.81	0.07	−0.05	15.98	−0.25	0.80
Participant 2	−0.17	42.74	−1.43	0.15	0.21	3315.33	1.91	0.05
Participant 3	0.03	38.50	0.21	0.84	0.24	19.35	1.29	0.20
Weighted average	0.03	0.11	0.28	0.78	0.27	0.15	17.98	0.07

Daily measure of symptom severity: degree of resistance to pulling urges.

A, baseline; B, treatment; A_1_, follow-up. The values in bold refer to significant changes in level of non-overlapping data between phases.

### Degree of Distress Associated With Pulling

Participants 1 and 2 reported low variability in distress associated with pulling during baseline, with relatively low and high distress, respectively. Participant 1 showed an increase during treatment and a sudden decrease at the end of the phase. There was high variability during the follow-up phase with no discernible pattern. No significant differences between phases emerged. Participant 2 exhibited a downward, but variable, trend in distress associated with pulling during the first weeks of treatment, but an increase in the last several weeks. There was a significant change in level of non-overlapping data between baseline and treatment. After the first few weeks of follow-up, distress dropped to a level of zero; nonetheless, no significant difference between phases emerged. Participant 3 demonstrated steadily increasing levels of distress associated with pulling across baseline. These levels then decreased steadily during treatment to no distress, followed by a stable report of low distress during follow-up. Significant changes in levels of non-overlapping data between baseline and treatment and treatment and follow-up emerged (Figure 2, Table 4).

**Table 4:** Tau-U calculations across phases for each participant.

	A vs. B + trend B − trend A				B vs. A_1_ + trend A_1_ − trendB			
Participant	Tau-U	SD Tau	*Z*	*p*	Tau-U	SD Tau	*Z*	*p*
Participant 1	0.35	14.39	1.60	0.11	−0.28	16.06	0.17	0.29
Participant 2	0.28	43.30	2.24	0.03	−0.17	54.04	−1.57	0.12
Participant 3	−0.49	39.32	−3.46	0.001	0.53	19.65	2.85	0.004
Weighted average	−0.04	0.11	−0.40	0.69	0.01	0.15	0.03	0.98

Daily measure of symptom severity: degree of distress associated to pulling.

A, baseline; B, treatment; A_1_, follow-up. The values in bold refer to significant changes in level of non-overlapping data between phases.

### Depression Anxiety Stress Scales-21

None of the participants endorsed clinically significant scores on the DASS-21 at baseline, nor reliable changes in their levels of distress from baseline to post-intervention (Table 5).

**Table 5:** DASS-21 scores for participants at baseline and post-treatment.

Baseline	Post-treatment	Outcome
12	3	No change
23	36	No change
1	4	No change

## Discussion

We used a multiple-baseline SCED design with three Italian women diagnosed with TTM to evaluate the efficacy of an eight-session ComB treatment that was designed to flexibly address the different factors maintaining hair-pulling by integrating cognitive and behavioral strategies using individualized case conceptualization (Mansueto et al., 1997, 1999; Falkenstein et al., 2016; Bottesi et al., 2019). All participants responded to some extent to the intervention, though none of them recovered fully. Each participant changed their hair-pulling behavior consistent with their clinical history and phenomenological features. In other words, the symptom measures on which they demonstrated improvements were those most relevant to the maintenance of their disorder. Tau-U calculations supported the presence of these changes. Of particular note, between-session exercise completion was 100%, and attendance and retention were excellent; none of the participants dropped out, highlighting the feasibility and acceptability of this treatment.

Participant 1 reported low levels of hair-pulling episodes, number of pulled eyelashes, and associated distress during the baseline period. The frequency of her episodes decreased over time, and she reported an increase in her ability to resist pulling urges during the intervention. This improvement was particularly important for this patient. Although her disorder had become less severe prior to participating in the study and she had managed to accept living with TTM over time, she continued to struggle with regulating negative emotions. Expanding her repertoire of strategies helped her improve her symptoms even further. Her increased ability to inhibit/interrupt hair-pulling was evident at Session 4, suggesting that she may have benefitted in particular from cognitive restructuring, behavioral experiments, and stress-management to modify the cognitive and emotional hair-pulling triggers and, consequently, reduce the hair-pulling behavior (Rehm et al., 2015b; Lootens and Nelson-Gray, 2016).

Participant 2 reported high levels of hair-pulling episodes (up to six episodes/day), number of hairs pulled, associated distress, and almost no resistance to pulling urges prior to treatment. Within the first three intervention sessions, she demonstrated improvements in number of pulling episodes (maximum one a day), associated distress, and ability to inhibit/interrupt hair-pulling behavior. These findings suggest that this patient may have benefitted from receiving accurate information about the disorder and better understanding the specific factors that helped maintain her hair-pulling. Indeed, these aspects may be a fundamental early treatment target given the frequency with which people with TTM report feelings of shame and isolation (e.g., Franklin and Tolin, 2007), negative beliefs about themselves and the disorder, and low self-efficacy about their coping ability (Rehm et al., 2015b). Indeed, Participant 2 endorsed an extremely negative attitude toward TTM when she entered the intervention. Improving her perceived coping ability may have contributed to her reduced distress (Franklin and Tolin, 2007). Identifying and implementing idiosyncratic behavioral control strategies may have played a crucial role in reducing her daily hair-pulling episodes (though the number of hairs pulled remained relatively stable during the treatment phase and in fact increased relative to baseline). Notably, this patient’s ability to resist hair-pulling urges improved during the follow-up phase. Following a severe and extremely distressing personal stressor between Sessions 3 and 4, all of her symptoms worsened. Given that her treatment gains occurred within a relatively short timeframe (3 weeks), and thus were not yet habitual, she almost completely resumed hair pulling in an attempt to manage negative affect. Moreover, although her DASS-21 score did not reliably deteriorate from baseline to post-treatment, her post-intervention level of general distress was clinically significant (*z* = 2.86), and we consequently referred her to a public outpatient psychiatric service.

Finally, Participant 3 reported a low number of hair-pulling episodes, but pulling a moderate number of hairs during baseline, along with difficulties inhibiting/interrupting her pulling behavior and increasing distress across that phase. During the intervention, she demonstrated reduced distress and number of hairs pulled per episode, and an increased ability to resist pulling urges. Participant 3’s increased ability to effectively interrupt the behavior may have contributed to the lower number of pulled hairs. Although there was a sudden reduction in this ability during the last weeks of the intervention, her hair-pulling episodes remained characterized by relatively few pulled hairs. During follow-up, Participant 3 reported having pulled her hair only during the 3 days preceding her final exam at university (which likely represented the most stressful situation she had to face). Although she pulled only two hairs per episode and associated distress was low, she reported five episodes a day. Despite the overall benefit she received from the intervention, she did not completely maintain her improvements, which is consistent with research highlighting difficulties maintaining treatment response in TTM (e.g., Falkenstein et al., 2014).

Taken together, our results support both the utility of delivering individualized psychological treatments for TTM and, ultimately, the ComB as a promising intervention given its flexibility. Furthermore, our single-case design allowed us to measure diverse behavioral changes when applying individualized and flexible psychological treatments, while taking into account potentially co-occurring variables that may confound clinical outcomes (Blampied, 1999; Kazdin, 2011). Future research investigating case conceptualization-driven treatments will likely benefit from this approach. Of note, no men responded to our recruitment advertisements. On the one hand, it may be that Italian cultural expectations of men lead them to be more reluctant to seek medical care than are women. On the other hand, the only study that focused on gender prevalence of TTM among Italian individuals reported that the female to male ratio was 14:1 (Bottesi et al., 2016b), which suggests that TTM is a disorder that, in Italy, affects mainly women. Therefore, we have to interpret our current findings with caution. Indeed, since triggers, emotional states, external environmental cues, cognition, and beliefs related to the onset and maintenance of TTM may be different in men and women, men may benefit from ComB treatment in a completely different way.

We must interpret our findings in the context of its limitations. First, this was a very small sample size. However, the primary goal of this pilot study was to evaluate the feasibility of this kind of treatment and provide initial estimates for its potential efficacy. The multiple-baseline design controls for the effects of time, while consuming minimal resources. Moreover, the small sample size allowed us to focus on each participant’s individual clinical characteristics, providing rich detail on potential mechanisms of change. Nonetheless, it is unclear whether our findings would generate to the larger population, including men. Given that our findings suggest the treatment is a promising option for people suffering from TTM, larger-scale studies with appropriate control groups are warranted. Second, we did not include a standardized measure of TTM symptom severity, so we could not calculate reliable and clinically significant change for each participant. Unfortunately, there is currently no Italian-language validated symptom questionnaire. The Italian Hair Pulling Questionnaire (Cerea et al., 2014) is the sole Italian measure designed to evaluate TTM, but it does not include a global severity index. Furthermore, we did not reassess either TTM symptoms or general distress severity (by readministering the SCID-5 and the DASS-21) at the 3-month follow-up. Nonetheless, daily self-monitoring is the typical primary outcome measurement method in SCEDs, because it allows for assessment of intraindividual changes across time. Third, because none of the three participants endorsed clinically significant levels of general distress (as measured by the DASS-21) at pre-treatment or reliable improvement on this variable post-treatment, one might argue that they were not psychologically impaired at the time of the intervention. However, all participants were formally assessed, and their symptoms met DSM-5 diagnostic criteria for TTM. TTM is a chronic disease, but its course fluctuates between exacerbations and remissions (Minichiello et al., 1994). It is possible that participants were not severely distressed at the time of the study; however, the impairment from their TTM was severe enough to warrant a clinical diagnosis. Fourth, none of the participants demonstrated full recovery or response maintenance, which raises issues about treatment duration. Mean duration of the disorder was 14.3 years, which means that it was a chronic condition: eight sessions were probably not sufficient to obtain complete recovery. As suggested by Falkenstein et al. (2014, 2016), patients may need to be treated until they are able to avoid hair-pulling altogether. In other words, the duration of a ComB treatment should be another flexible and individualized treatment component. Fifth, none of the patients engaged in self-monitoring frequently during the follow-up phase. It is unclear whether this was attributable to lower engagement with self-monitoring (because of reduced study requirements during that period) or to a reduced frequency of episodes. Indeed, participants may have been monitoring less frequently because there was less to monitor. It may be important in future research and/or during intervention to encourage patients to self-monitor every day, even if their report is zero hair-pulling for that day. Finally, we cannot exclude the possibility that at least some of the noted improvements were due to placebo effects. The treatment phase, in addition to containing active components of therapy, also contained the “common factors” of therapy – including contact with a caring clinician, an opportunity to discuss the problem, emotional arousal, expectancy/faith/hope in the therapist’s abilities as well as in the treatment, the provision of a rationale, etc. All of these factors could have accounted for the observed change in symptoms.

We recommend future research testing the efficacy/effectiveness of the ComB treatment for TTM using larger SCEDs and RCTs specifically in Italian clinical populations, while reducing the limitations noted above. Indeed, researchers have proposed other CBT interventions (dialectical behavior therapy, metacognitive therapy, and acceptance and commitment therapy), for TTM (e.g., Keuthen et al., 2012; Lee et al., 2018; Shareh, 2018); future studies comparing these with the ComB treatment are warranted. As such, flexibility may extend from individualized case conceptualizations to selection of treatment option, offering multiple avenues for recovery to people with TTM.

## Data Availability Statement

The raw data supporting the conclusions of this article will be made available by the authors, without undue reservation.

## Ethics Statement

The studies involving human participants were reviewed and approved by Ethical Committee for the Psychological Research of the University of Padova. The patients/participants provided their written informed consent to participate in this study. Written informed consent was obtained from the individual(s) for the publication of any potentially identifiable images or data included in this article.

## Author Contributions

GB, CS and MG contributed conception and design of the study. EC organized the database. UG performed the statistical analysis. GB wrote the first draft of the manuscript. AO and SC wrote sections of the manuscript. All authors contributed to manuscript revision, read, and approved the submitted version.

## Conflict of Interest

GB, SC, and MG declare that they might receive book royalties on closely related topics.

The remaining authors declare that the research was conducted in the absence of any commercial or financial relationships that could be construed as a potential conflict of interest.

## Footnotes

^1^https://clinicaltrials.gov/ct2/show/NCT03486041
